# Development of a Computerized Adaptive Test for Quantifying Chinese Medicine Syndrome of Myasthenia Gravis on Basis of Multidimensional Item Response Theory

**DOI:** 10.1155/2021/9915503

**Published:** 2021-05-24

**Authors:** Zhongyu Huang, Yunying Yang, Fengbin Liu, Lijuan Li

**Affiliations:** ^1^Integrated Chinese and Western Medicine Postdoctoral Research Station, Jinan University, Guangzhou 510632, China; ^2^The First Affiliated Hospital of Guangzhou University of Chinese Medicine, Guangzhou 510000, China; ^3^Institute of Pediatrics, Guangzhou Women and Children's Medical Center, Guangzhou Medical University, Guangzhou, China

## Abstract

**Background:**

Making comprehensive management of myasthenia gravis (MG) is a challenge in clinical practice due to heterogeneity and multiple comorbidities among patients.

**Aim:**

To develop an end-to-end instrument for individualized assessment of MG in the perspective of Chinese medicine (TCM) with the application of multidisciplinary quantification approaches.

**Methods:**

A self-administrated questionnaire was developed integrating typical symptoms of MG and spleen-kidney deficiency syndrome on basis of the conceptual framework of TCM. With data collected in a multicenter cross-sectional study, confirmatory factor analysis together with multidimensional item response theory (MIRT) was used for evaluating the psychometric property of the questionnaire. A computerized adaptive test was developed based on the MIRT model, and scores of syndrome factors were calculated in simulation. A logistics regression model was also estimated for evaluating the consistency between the quantitative result and the clinical diagnosis of syndrome from clinical practitioners.

**Result:**

With 337 patients enrolled and assessed, the 14-item questionnaire was evaluated to be with adequate validity and reliability (Cronbach's alpha indices = 0.87, AIC = 195.827, BIC = 348.631, CFI = 0.921, RMR = 0.006, GFI = 0.954, RMSEA = 0.048, and *χ*2/df = 1.782). With adequate factor loadings of symptoms on related syndrome factor, the instrument was evaluated with preliminary interpretation and was suitable for evaluating patients with moderate severity of the spleen and kidney deficiency syndrome.

**Conclusion:**

Setting typical symptoms of MG together with systemic discomforts in a computerized adaptive test on the basis of MIRT, this study proposed an innovative research paradigm for quantifying individual condition in the perspective of TCM with application of interdisciplinary approaches.

## 1. Background

As an autoimmune neuromuscular disease, myasthenia gravis (MG) was reported to be mediated by autoantibodies targeting components of the neuromuscular junction [1]. Typical appearances of muscle weakness such as eyelids droops and fatigue result in reduction of daily activity and negtively affect the quality of life of patients. Restrictive respiratory failure caused by severe weakness of respiratory muscle could even lead to emergency known as MG crisis in 15% of MG patients [2]. The heterogeneity of clinical appearances of MG ranging from mild ocular deficits to severe widespread weakness posed a challenge for clinical assessment of MG patients [3]. Moreover, multiple comorbidities among patients with chronic disease were also commonly reported which made it difficult for the management of MG. Due to the disease heterogeneity, MG is increasingly acknowledged as a syndrome more than a single disease [4]. The goal of treatment of MG is to obtain remission and disease stability with the least symptoms and that was thought to be a challenge [5]. It is critical to introduce ideas and approaches from chronic disease management and develop instrument for comprehensive measurement and individualized monitoring of patients with MG [6].

Quantitative instruments were developed as assistive tools for individual assessment of MG patients [7]. For example, the Myasthenia Gravis Score [8] and the Myasthenia Gravis Composite [9] were developed and used for measuring the clinical outcome of MG by quantifying disease severity. The myasthenia gravis patient-reported outcome scale was developed for evaluating the quality of life of MG patients and supporting measurement of treatment effects in clinical trials about MG [10, 11]. These instruments served as practical tools for the management of MG offering quantitative scores as references for clinical diagnosis and decision of treatment. However, shortcomings of these scales are also obvious among which the lengthy setting of scalesalways resulted in reduction of compliance of patients during assessment. Moreover, the interpretability of the traditional assessment strategy was impaired since much information was lost while accumulating the scores with compensatory logic. The “one size fits all” approaches were reported to be without relevance. And individualized diagnosis and treatment approaches are required to match the heterogeneity of MG patients.

Traditional Chinese medicine (TCM) practitioners pursue individualized diagnosis and therapy by summarizing symptoms and signs of patients within the conceptual framework of syndrome differentiation. Falling within the scope of Flaccidity Syndrome in TCM theory, MG was known to be caused by deficiency of spleen. As a complementary and alternative medical approach, TCM therapies with herbs and acupuncture were reported to help releasing severity of muscular fatigue and improving quality of life of MG patients [12–18]. Pharmacological effects and pathogenesis of MG were also explored with the application of statistical and machine learning methods in the perspective of TCM [19, 20]. However, controversy remained about the abstract theory and empirical practice of TCM. Innovative research strategies should be established for measuring the efficacy of TCM therapy and further strengthening the interpretability of TCM theory.

With the purpose of quantifying abstract concepts in TCM theory, many innovative research paradigms were proposed with application of interdisciplinary methods including structural equation modeling (SEM) and multidimensional item response theory (MIRT) [21, 22]. In these studies, mathematical models were estimated bridging the gap between observable symptoms or signs and syndromes which were regarded as the latent trait of patients.In this way, individual condition could be quantified within a interpretable conceptual framework. With application of computer science and information technology, the traditional form of assessment was also shifted into a more efficient mode and that further enabled individualized evaluation on basis of quantitative model [23–25].

Aiming at providing a flexible approach to support clinical management of MG patients in the perspective of TCM, this article proposed an innovative strategy for quantifying TCM syndrome of MG with development of a computerized adaptive test (CAT) on the basis of MIRT.

## 2. Method and Materials

### 2.1. Data Source

A multicenter cross-sectional study was carried out in China from Jun 2008 to Aug 2013. Diagnosis criteria of MG was set referring to guidance from the Handbook of Clinical Neurology [26]. Patients diagnosed as MG in age between 14 and 75 were recruited from three research institutions including the First Affiliated Hospital of Guangzhou University of Chinese Medicine, the Guangzhou Second People's Hospital, and Guangdong Province Hospital of Chinese Medicine after informed consent. Patients aged less than 14 or over 75 and those with malignant thymoma or serious comorbid diseases such as renal failure or psychiatric diseases were excluded. Pregnant or breast-feeding women with MG were also kept out in this study. All patients diagnosed with spleen deficiency syndrome or spleen-kidney deficiency syndrome were asked to fill the self-administrated questionnaire and those who did not complete the assessment were excluded for further analysis.

### 2.2. Questionnaire and Conceptual Framework of TCM Syndrome Assessment

Aiming at quantifying the severity of TCM syndrome about MG, the instrument was designed under the conceptual framework of TCM theory. The self-administrated questionnaire consisted of two parts. Firstly, an introduction about the purpose of the assessment together with fields of individual information such as name, gender, and age was formed at the top of the scale. Secondly, items describing typical symptoms or signs of MG served as the major part of the assessment for evaluating the syndrome severity of MG patients.

According to previous reports about the prevalence of syndromes about MG, deficiency of both spleen and kidney was known as major pathogenesis of MG in TCM theory [27, 28]. Therefore, the conceptual framework of the instrument was set limiting the scope of assessment over major syndromes including spleen deficiency syndrome and spleen-kidney deficiency syndrome. A set of symptoms or signs as clues for syndrome differentiation was listed and discussed and then transformed into items with dichotomous options. A group of 3 clinical experts was invited taking the responsibility of evaluating and validating the content description and option settings of the items so as to ensure the rationalization of the questionnaire considering both clinical fitness and cultural competence.

**Figure 1:**
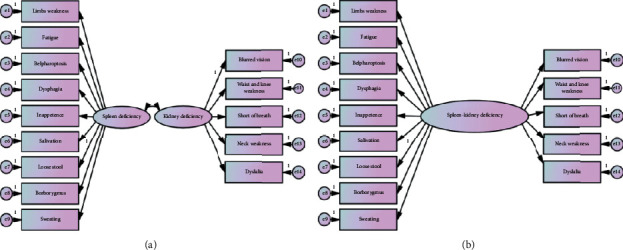
Conceptual framework of spleen-kidney deficiency syndrome of myasthenia gravis. (a) Multidimensional model of spleen-kidney deficiency syndrome. (b) Unidimensional model of spleen-kidney deficiency syndrome

Conceptual framework of spleen-kidney deficiency syndrome was shown in Figure 1(a) and items consisted of the framework were listed in Table 1. There were 14 typical symptoms in the framework among which 9 items were drafted for quantifying spleen deficiency including weakness of limbs, fatigue, blepharoptosis, inappetence, dysphagia, salivation, loose stool borborygmus, and sweating with the other 5 items for quantifying kidney deficiency items including weakness of waist and knee, weakness of neck, dyslalia, shortness of breath, and blurred vision. The instrument was reviewed and approved by the chief of experts before promoting the assessment.

During the assessment, demographic details of the examinees including gender, age, occupation, and education were recorded in the first section of the questionnaire. Patients were also asked to fill in the items following the introduction of the professional clinical practitioner. And the practitioner took the responsibility of explaining the content to reduce misunderstanding of the items. Initial opinion of syndrome differentiation was made by a trained practitioner after the assessment. And another practitioner with senior title was in charge of making confirmation of the diagnosis. Once there was a conflicting idea that occurred toward the clinical diagnosis of the syndrome, a third practitioner with senior title would be invited to make a discussion for the final decision.

### 2.3. Development of CAT on Basis of the MIRT Model

As all the responses of the items were collected from patients, the 2-parameter logistic model was used for estimating the psychometric parameters of items. And quasi-Monte Carlo Expectation-Maximum (EM) estimation was used as the estimation algorithm. As to the parameters setting of the estimation, a limitation was also set with the maximum number of EM cycles as 2000 and the standard error tolerance criteria for the computation of the information matrix as 0.001. On basis of the MIRT model, the CAT was created with package mirtCAT [29] in *R* 3.6.2 and the logic of assessment was designed as follows: (i) Starting item was randomly selected in range of major appearances of MG including blepharoptosis, fatigue, and weakness of limbs. (ii) The maximum determinant of the information matrix was set as adaptive criteria of the assessment for the latent trait scores calculation. (iii) Stopping criteria of CAT were set with the delta of latent trait scores as 0.05 and the minimum standard error of each dimension as 0.3. Furthermore, a web-based questionnaire was designed offering an interactive interface for the assessment. As far as compliance of patients was concerned, the CAT assessment was carried out in simulation with the response of the original assessment.

### 2.4. Statistical Analysis

Descriptive analysis about demographic characteristics was carried out in SPSS 22.0. To evaluate the validity of the scale, Cronbach's alpha indices were analyzed in SPSS 22.0. The psychometric property of the items was evaluated with the assistance of package mirt in *R* 3.6.2 [30]. Construct validity of the multidimensional and unidimensional model shown in Figures 1(a) and 1(b) were evaluated referring to indices estimated in Confirmatory Factor Analysis (CFA) including Root Mean Square Residual (RMR), Root Mean Square Error of Approximation RMSEA), Comparative Fit Indices (CFI), and Goodness of Fit Indice (GFI). Multidimensional discrimination index (MDISC) and multidimensional difficulty index (MDIFF) of each item were also evaluated indicating reliability of item setting. Item information surface of each item was plotted as intuitive visualization of the property of the items and the entire assessment. With a CAT developed in *R* 3.6.2, multidimensional traits of each patient were estimated in stimulation. Setting clinical diagnosis of syndrome as reference, Receiver Operation Curve (ROC) was estimated and the area under the curve (AUC) was calculated in *R* 3.6.2 for evaluating the accuracy of the model.

## 3. Result

### 3.1. Demographic Analysis

As shown in Table 2, a total number of 337 patients were finally enrolled in this study with 12 cases excluded out of the unfinished assessment. Male took a larger percentage than female and the elder was less than the young and mid-age patients. The mean age of the sample was 37.947 ± 16.358 and the patients in youth and middle age took up a major proportion as 58.46% and 29.67% of the sample. Ranking with the frequency of clinical appearances as shown in Table 3, typical symptoms including blepharoptosis, weakness in limbs, fatigue, and dysphagia were most frequently reported and that is consistent with previous reports [31]. It should be noticed that systemic symptoms such as inappetence and shortness of breath were also commonly reported and that could be important factors influencing the quality of life of MG patients.

As to the validity and reliability of the instrument, consistency of the response of items in the assessment was evaluated to be adequate with Cronbach's alpha indices as 0.87. Split-half validity was also calculated in an acceptable condition as 0.87. The goodness of fit about the conceptual framework as construct validity of the instrument was also evaluated to be adequate with AIC = 195.827, BIC = 348.631, CFI = 0.921, RMR = 0.006, GFI = 0.954, RMSEA = 0.048, and *χ*2/df = 1.782. As comparison, the fitness indices of the unidimensional model were estimated with AIC = 286.537, BIC = 393.500, CFI = 0.762, RMR = 0.009, GFI = 0.908, RMSEA = 0.077, and *χ*2/df = 2.99.

**Figure 2:**
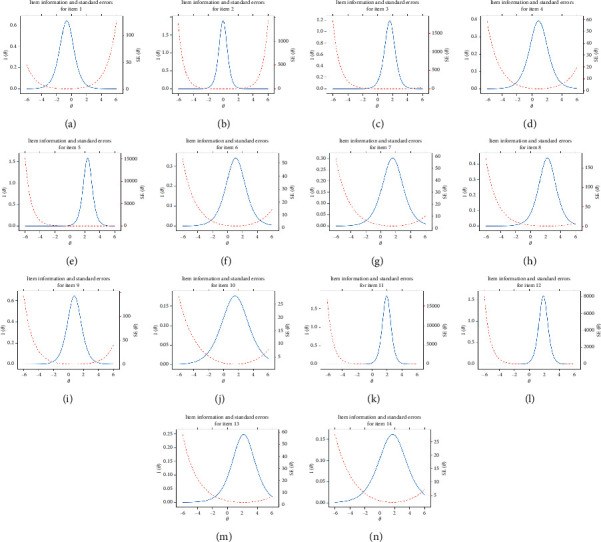
Item information and standard errors curves of the 14 items in the questionnaire.

**Figure 3:**
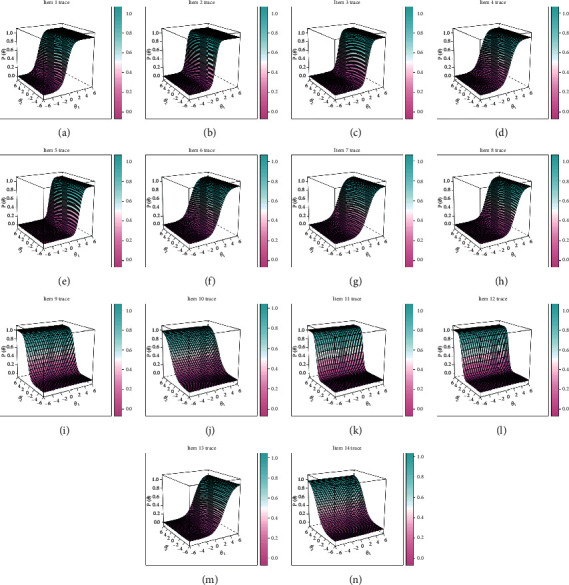
Trace surfaces of the 14 items in the questionnaire.

**Figure 4:**
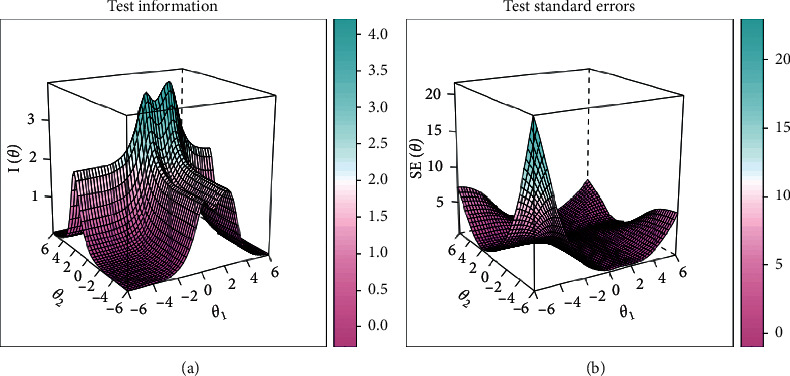
Information and standard error surfaces about the test with the 14-item questionnaire.

Psychometric parameters including MDISC, MDIFF, and standardized factor loading were estimated with MIRT and shown in Table 4. All items were evaluated with adequate discrimination for assessment with MDISC over 0.5. Information characteristics and standard error curves of the items were plotted and shown in Figure 2. The setting of all items was evaluated to be adequate as most information and least standard error could be achieved for those with a moderate score of latent traits. And the trace surfaces showed in Figure 3 indicated that items were with adequate setting to discriminate patients in different severity. Moreover, with both latent traits scores in range (−2, 2), most information and least standard error could be achieved as the humps of the information surface shown in Figure 4. Settings about items of the instrument were evaluated to be proper therefore ensuring the assessment with adequate validity and reliability.

Factor loadings of symptoms on their related latent factor were also evaluated to be consistent with the conceptual setting. For spleen deficiency syndrome factor, fatigue and digestive discomforts such as dysphagia took a loading value over 0.5 as shown in Table 4. As far as kidney deficiency was concerned, shortness of breath and dyslalia together with the weakness of waist and knees took the highest loadings as 0.856, 0.823, and 0.712. The information surface and standard error of the test shown in Figure 4 showed that most information would be achieved for examinees with severity of both dimensions in a moderate range in (−3, 3).

As psychometric parameters of the items were estimated, the CAT was developed for individualized assessment of TCM syndrome of MG. Latent traits of patients were estimated with a stimulated assessment with multidimensional scores in the range of (−6, 6). Correlation between clinical diagnosis and latent trait scores was evaluated to be significant in logistics regression. Results of the regression analysis showed that the two latent traits were calculated to be statistically significant correlated with the clinical diagnosis of syndrome with the correlation coefficients as 2.088 (*p* < 0.01) and 6.593 (*p* < 0.01) as shown in Table 5.

**Figure 5:**
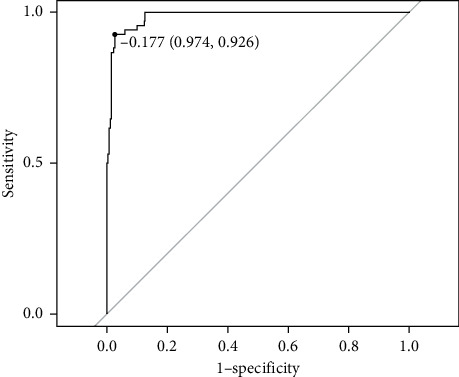
Receiver operating characteristic curve about the computerized adaptive test on basis of multidimensional model.

AUC was evaluated as 0.986 indicating that the predicted score was in adequate consistency with the clinical diagnosis of syndrome. Best performance with sensitivity as 0.926 and specificity as 0.974 could be achieved while setting the threshold of the model as −0.177 as shown in Figure 5.

## 4. Discussion

In this study, female preponderance was found with the male:female ratio evaluated as 1 : 1.21. And that is similar with studies reported in other regions of the world. [32, 33]. Although patients in different range of age took different corporation of the sample, no significant conclusion could be drawn because neither the onset time nor the duration information but only the attendance time was exactly recorded. While tracing back the development of MG, the diagnosis procedure of patients always lasted long and patients intended to seek treatment with TCM as an alternative approach.

Heterogeneity of MG patients in different gender, ages, duration of disease, and more importantly the comorbidity with different diseases made it a challenge to make comprehensive management of MG patients. As shown in Table 4, besides the most commonly reported symptoms, systemic appearances such as inappetence were calculated with frequency not lower than typical MG appearances. It should be noticed that these discomforts could be caused by multifactors including side effects of drugs. Therefore, it is a challenge to make a comprehensive interpretation of the clinical appearances of MG patients.

Standardized rules of diagnosis and treatment are important while individualized management is also essential to meet different requirements of patients. In the clinical practice of TCM, syndromes were concluded as summarization of systemic appearances in a conceptual framework. Accordingly, therapies were then designed to adjust the individual status with balance therefore achieving the goal for relieving the severity of all symptoms. Spleen-kidney deficiency syndrome was reported to be the major syndrome of MG patients in China. [16, 34]. In TCM theory, the kidney governs the bone and acts as the root of primordial Qi to dominate growth and development. And deficiency of primordial Qi directly influences growth, development, and muscular function. The transformation function of spleen provided nutrients for muscle and energy metabolism mainly relying on the transporting of Qi. Therefore, deficiency of spleen and kidney leads to failure in transporting food and nutrients that caused digestive disorders symptoms such as belching and loss of appetite. That further leads to disorder in nourishing muscle and makes muscles atrophied and become asthenic resulting in symptoms involving ocular, bulbar, respiratory and proximal limb muscles [28]. Following the conceptual framework of TCM, the model of assessment was conducted with two latent factors including spleen deficiency and kidney deficiency. Related symptoms were drafted as clues for differentiation of each dimension of syndrome factors. Setting typical symptoms of MG together with systemic discomforts in a uniformed baseline, the paradigm of the study was delighted by the idea of syndrome differentiation in TCM theory.

Interdisciplinary approaches also benefit us with the possibility to develop individualized approaches to assist the management of MG. We proposed an innovative end-to-end strategy with development of an individualized assessment for MG in this article not only meeting the requirement of chronic disease management but also out of the exploration about the modernized clinical practice of TCM. The quantitative syndrome differentiation model under the conceptual framework of disease-syndrome integration covered the most important clinical appearances for analyzing the major pathogenesis of MG and was evaluated with adequate consistency with the clinical diagnosis of syndrome. With psychometric property of the items estimated in MIRT, severity of spleen deficiency and kidney deficiency as latent traits of patients was quantified with different clinical appearances in combination. Setting all of the items in a standardized scoring procedure, the CAT on basis of MIRT model equipped designer with adaptive logic of assessment meeting the requirement of different situations. Uncompensated scoring algorithm also makes it more suitable for the individualized evaluation of patient with complex clinical appearances. The logistic regression model of spleen and kidney deficiency was also evaluated with adequate accuracy with AUC evaluated to be 0.925 referring to the clinical diagnosis of syndrome. And the regression model bridged the gap between the assessment and decision of syndrome differentiation in this way making the CAT an end-to-end instrument.

To our knowledge, this is the first study that proposed a multidisciplinary paradigm for quantifying TCM syndromes of MG with application of multidimensional latent traits analysis and computerized adaptive testing. However, there are several limitations in our research. Firstly, since the research region was limited in Guangdong province, there was much uncertainty about the representativity of the sample although the patients were enrolled from a multicenter study. Secondly, there could be information loss and bias introduced from the assessment due to the rough dichotomous responses recorded with the instrument. Design of the instrument should be modified by setting the items with graded options and extending the scope of assessment for other syndromes of MG besides spleen-kidney deficiency syndrome. Thirdly, research should be carried out for estimation and evaluation of the stability, rationality, and further extrapolation of the model with a representative sample before further application in clinical practice. Last but not the least, controversies still exist either about the complex concepts in TCM theory or the empirical strategy in its clinical practice for the lack of objective evidence as practical clues for diagnosis and clinical decision of treatment. Further research should be carried out focusing on the estimation of standardized criteria for syndrome diagnosis and therapy in place of the traditional empirical approach for the modernized practice of TCM.

## 5. Conclusion

The establishment of instruments with interdisciplinary approaches for quantification and management of chronic and rare diseases such as MG would benefit the patients with continuous monitoring of individual condition and further promoting efficiency of treatment and management of disease. Setting typical symptoms of MG together with systemic discomforts in a uniform quantification baseline in the perspective of TCM, this study provided an innovative research paradigm to assist individualized management of MG with application of multidisciplinary approaches including MIRT and CAT.

## Figures and Tables

**Table 1:** Items of questionnaire about spleen-kidney deficiency syndrome of myasthenia gravis.

Index	Content	Option 1	Option 2	Related factor
1	Weakness of limbs	Yes	No	Spleen deficiency
2	Fatigue	Yes	No	Spleen deficiency
3	Blepharoptosis	Yes	No	Spleen deficiency
4	Inappetence	Yes	No	Spleen deficiency
5	Dysphagia	Yes	No	Spleen deficiency
6	Salivation	Yes	No	Spleen deficiency
7	Loose stool	Yes	No	Spleen deficiency
8	Borborygmus	Yes	No	Spleen deficiency
9	Sweating	Yes	No	Spleen deficiency
10	Weakness of waist and knees	Yes	No	Kidney deficiency
11	Weakness of neck	Yes	No	Kidney deficiency
12	Dyslalia	Yes	No	Kidney deficiency
13	Short of breath	Yes	No	Kidney deficiency
14	Blurred vision	Yes	No	Kidney deficiency

**Table 2:** Demographics and characteristics of the 337 myasthenia gravis patients.

Variables	Total (*n* = 337)	Proportion (%)
*Age*		
** **Youth (14–44)	197	58.46
** **Mid-age (45–59)	100	29.67
** **Elder (60–75)	40	11.87

*Gender*		
** **Female	185	54.89
** **Male	152	45.11

*Syndrome*		
** **Spleen and kidney deficiency	68	20.18
** **Spleen deficiency	269	79.82

**Table 3:** Frequency of symptoms reported with the 337 myasthenia gravis patients.

Item	Frequency	Percentage (%)
Blepharoptosis	286	84.87
Weakness of limbs	226	67.06
Fatigue	173	51.34
Dysphagia	158	46.88
Blurred vision	110	32.64
Inappetence	102	30.27
Weakness of waist and knee	101	29.97
Dyslalia	100	29.67
Salivation	91	27.00
Shortness of breath	85	25.22
Weakness of neck	83	24.62
Loose stool	66	19.58
Sweating	47	13.95
Borborygmus	30	8.90

**Table 4:** Estimated properties of the spleen-kidney syndrome model of myasthenia gravis.

Items	MDISC	MDIFF	Factor loading	
			Spleen deficiency	Kidney deficiency
Weakness of limbs	1.587	−0.641	0.682	0
Fatigue	2.841	−0.018	0.858	0
Blepharoptosis	2.177	1.612	0.788	0
Inappetence	1.263	0.870	0.596	0
Dysphagia	2.423	2.420	0.818	0
Salivation	1.141	1.097	0.557	0
Loose stool	1.093	1.581	0.54	0
Borborygmus	1.287	2.262	0.603	0
Sweating	0.999	2.142	0.506	0
Weakness of waist and knees	1.725	0.743	0	0.712
Weakness of neck	0.841	1.522	0	0.443
Dyslalia	2.463	1.989	0	0.823
Shortness of breath	2.816	1.790	0	0.856
Blurred vision	0.828	1.673	0	0.437

**Table 5:** Estimated parameters of logistics regression model with multidimensional scores from the computerized adaptive test.

Variable	Estimate	Standard error.	z-value	Pr (>|z|)
(Intercept)	−5.322	0.907	−5.866	0.000
Spleen deficiency	2.088	0.637	3.279	0.001
Kidney deficiency	6.593	1.145	5.760	0.000

## Data Availability

The data used to support the findings of this study are available from the corresponding author (e-mail: jlily0252@126.com) upon request.

## Abbreviations

TCM: Traditional Chinese medicine
MG: Myasthenia gravis
CFA: Confirmatory factor analysis
MIRT: Multidimensional item response theory
FGIDs: Functional gastrointestinal disorders
SEM: Structural equation modeling
CAT: Computer adaptive test
CFI: Comparative fit indices
GFI: Goodness of fit indices
RMSEA: Root mean square error of approximation
RMR: Root Mean Square Residual
AIC: The Akaike information criterion
*χ*^2^: Chi-square
Df: Degree of freedom
MDISC: Multidimensional discrimination index
MDIFF: Multidimensional difficulty index
IIS: Item Information Surface
EM: Expectation-Maximum
ROC: Receiver operation curve
AUC: Area under the curve.
